# Executive summary for the Micronutrient Powders Consultation: Lessons Learned for Operational Guidance

**DOI:** 10.1111/mcn.12493

**Published:** 2017-09-29

**Authors:** Christina Nyhus Dhillon, Danya Sarkar, Rolf DW Klemm, Lynnette M Neufeld, Rahul Rawat, Alison Tumilowicz, Sorrel ML Namaste

**Affiliations:** ^1^ Independent Consultant Geneva Switzerland; ^2^ Strengthening Partnerships, Results, and Innovations in Nutrition Globally Arlington Virginia USA; ^3^ John Snow, Inc. Arlington VA USA; ^4^ Helen Keller International Washington District of Columbia USA; ^5^ Johns Hopkins Bloomberg School of Public Health Baltimore Maryland USA; ^6^ Global Alliance for Improved Nutrition Geneva Switzerland; ^7^ International Food Policy Research Institute Dakar Senegal; ^8^ Bill and Melinda Gates Foundation Seattle Washington USA

**Keywords:** complementary feeding, evidence‐based practice, infant and child nutrition, iron deficiency anaemia, micronutrients, programming

## Abstract

Iron deficiency anaemia is estimated to be the leading cause of years lived with disability among children. Young children's diets are often inadequate in iron and other micronutrients, and provision of essential vitamin and minerals has long been recommended. With the limited programmatic success of iron drop/syrup interventions, interest in micronutrient powders (MNP) has increased. MNP are a mixture of vitamins and minerals, enclosed in single‐dose sachets, which are stirred into a child's portion of food immediately before consumption. MNP are an efficacious intervention for reducing iron deficiency anaemia and filling important nutrient gaps in children 6–23 months of age. As of 2014, 50 countries have implemented MNP programmes including 9 at a national level. This paper provides an overview of a 3‐paper series, based on findings from the “Micronutrient Powders Consultation: Lessons Learned for Operational Guidance” held by the USAID‐funded Strengthening Partnerships, Results, and Innovations in Nutrition Globally (SPRING) Project. The objectives of the Consultation were to identify and summarize the most recent MNP programme experiences and lessons learned for operationalizing MNP for young children and prioritize an implementation research agenda. The Consultation was composed of 3 working groups that used the following methods: deliberations among 49 MNP programme implementers and experts, a review of published and grey literature, questionnaires, and key informant interviews, described in this overview. The following articles summarize findings in 3 broad programme areas: planning, implementation, and continual programme improvement. The papers also outline priorities for implementation research to inform improved operationalization of MNP.

## 1 INTRODUCTION

Globally, 43% or 300 million children under five are anaemic, with the greatest burden of the disease in Africa and Asia, and trends have shown only a slow decline from 47% in 1995 (Stevens et al., 2013). Although there are many causes of anaemia, iron deficiency is the most common nutritional cause (Khambalia & Zlotkin, 2003; World Health Organization, 2001). Anaemia has significant adverse health consequences and negative impacts on social and economic development (Balarajan, Ramakrishnan, Ozaltin, Shankar, & Subramanian, 2011, 2013). Iron deficiency (with or without anaemia) during early childhood is associated with impaired motor and mental development, poorer socio‐emotional behaviour, and reduced school achievement (Lozoff et al., 2006). Because of these negative consequences, iron deficiency anaemia (IDA) is estimated to be the leading cause of years lived with disability among children (Global Burden of Disease Pediatrics Collaboration et al., 2016).

IDA is of particular concern during infancy, as iron requirements are relatively high during periods of rapid growth (Stoltzfus, Mullany, & Black, 2004). In young children, peak prevalence of IDA occurs around 18 months, then falls, as iron requirements decline and iron intake is increased through complementary foods (Black et al., 2008). Iron deficiency is not the only common micronutrient deficiency in young children, and multiple micronutrient deficiencies are often concurrent. Deficiencies in vitamin A, iodine, zinc, and other micronutrients are also significant public health issues (Ramakrishnan, 2002). Meeting the micronutrient needs of children 6–23 months of age is critical, yet it is difficult to ensure young children obtain an adequate quantity and quality of complementary foods containing these nutrients. A nutrient‐dense diet is needed to meet these requirements within the limited volume of food children consume (Brown, Dewey & Allen, 1998; Dewey, 2013). This high nutrient density can be challenging to achieve especially in low resource settings (Yip, Binkin, Fleshood, & Trowbridge, 1987). Even when micronutrient‐rich complementary foods are available and optimized, it is still often necessary to complement the diet using specific interventions, such as food fortification or supplementation (Bhutta, Salam, & Das, 2013; Osendarp et al., 2016). One such point‐of‐use intervention are micronutrient powders (MNP)—a mixture of vitamins and minerals, enclosed in single‐dose sachets, which are stirred into a child's portion of food immediately before consumption.

Under controlled conditions in low‐income countries, MNP containing between five and 15 nutrients have been shown to be efficacious at reducing iron deficiency by 51% and anaemia by 31% in children under 2 years of age, regardless of anaemia prevalence and duration of dosing (intervention duration from 2 to 12 months) (De‐Regil, Suchdev, Vist, Walleser, & Peña‐Rosas, 2013). Although studies examining the impact of MNP on nutritional problems beyond iron deficiency are limited, there is some evidence that MNP may reduce vitamin A deficiency (Suchdev et al., 2012) and stunting (Rah et al., 2012; Shafique et al., 2016; Soofi et al., 2013). MNP have generally replaced iron drops/syrups as the preferred intervention given the research showing similar efficacy but higher acceptability and fewer side effects (Dewey, Yang, & Boy, 2009). The World Health Organization recommends the use of MNP containing iron, vitamin A, and zinc with or without other micronutrients to achieve 100% of the recommended nutrient intake for children 6–23 months of age (World Health Organization, 2011, 2016) and more recently has expanded this recommendation for its use in children 2–12 years of age (World Health Organization, 2016). The guidelines state that these recommendations are to be implemented in the context of programmes aimed at improving infant and child health and nutritional status.

**Key Messages**

- Micronutrient powders, an efficacious intervention to address iron‐deficiency anaemia and potentially other micronutrient deficiencies in young children, appear simple but are quite complex to implement.
- A wide variety of experiences implementing micronutrient powders exist‐there is no one size fits all approach‐but select generalizable lessons can be distilled.
- Despite growing evidence on approaches to plan, deliver and monitor programs, more needs to be understood, particularly around sustaining and scaling the intervention. Currently micronutrient powders pilots and subnational implementation still predominate. More scaled interventions need documentation of program learning.

### 1.1. Rationale for consultation

Three benefits to MNP are as follows: (a) They are efficacious in reducing both anaemia and iron deficiency in children; (b) they are easy to use and do not require dietary change; and (c) they can be produced in large quantities at a relatively low cost (0.02$ per sachet) (de Pee et al., 2008). Recently, there has been global scale‐up of MNP interventions—predominantly in the regions of Asia and Africa—increasing from 36 interventions in 22 countries to 59 interventions in 50 countries between 2011 and 2014, according to the NutriDash survey administered to United Nations Children's Fund (UNICEF), government, non‐government organizations, and other partner staff in 159 countries (Jefferds, Irizarry, Timmer, & Tripp, 2013; UNICEF, 2015). Of these 50 countries, 9 were implementing national and 20 subnational programmes (UNICEF, 2015). Despite the rapid adoption, the extent to which the quality and scalability of MNP interventions can be maintained has yet to be well established (Rah et al., 2012).

In July 2015, the Strengthening Partnerships, Results, and Innovations in Nutrition Globally (SPRING) Project, on behalf of United States Agency for International Development (USAID), convened a year‐long consultation of policy makers, programme implementers, donors, and global experts to share evidence and experiences related to MNP interventions. The aim of the Consultation was to provide contextualized operational guidance in implementing MNP interventions for young children. The three objectives of the MNP Consultation were to (a) *identify and summarize experiences*, using existing documents, reports, and country experiences, with an emphasis on lessons learned from the field within MNP programming; (b) *define essential components* that should be included in any MNP programme to ensure national ownership, context specificity, and sustainability; and (c) prioritize an MNP *implementation research* agenda.

This supplement consolidates recent evidence about implementing MNP in order to provide relevant information for all stakeholders considering starting, scaling, or refining an MNP intervention and to support an implementation research agenda to be incorporated into programme and policy activities. The Consultation and its outcomes are intended to complement existing resources and tools for MNP interventions, predominantly from the Home Fortification Technical Advisory Group (HF‐TAG).

## 2 METHODS

### 2.1 Working groups

**Figure 1:**
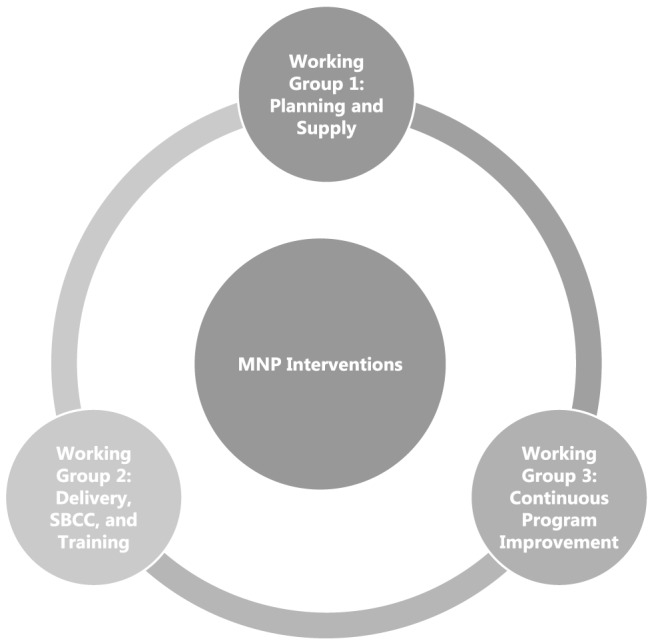
MNP Consultation working group components. The three working groups included (a) planning and supply; (b) delivery, social and behavioural change communication (SBCC), and training; and (c) monitoring, process evaluation, and supportive supervision for continuous programme improvement. The working groups were composed of country‐ and global‐level MNP implementers, and each working group summarized evidence and lessons learned in their respective programmatic area. MNP, micronutrient powders

A group of 49 experts, from 19 countries and 25 organizations, with experience in MNP interventions were invited to join the Consultation to discuss key lessons learned and operational challenges (Supporting Information S1). In July 2015, three working groups covering broad programme components—planning and supply (WG1); delivery, social and behaviour change communication (SBCC), and training (WG2); and monitoring, process evaluation, and supportive supervision for continual programme improvement (WG3) (Figure 1)—were formed to summarize evidence and lessons learned. Working groups were composed of country and global level implementers. A chair was identified for each working group, and the members communicated via telephone and email on a regular basis, details of which are further elaborated in each individual paper. On October 19 and 20, 2015, a face to face meeting in Washington, DC allowed for wider deliberation of the working groups' initial findings (SPRING, 2015).

The focus of the consultation was on programmes that delivered MNP to children 6–23 months of age, which was the population recommended by World Health Organization at the time of the consultation (2011). However, as the consultative process unfolded, learnings from pilots and programmes with a wider target age (up to 59 months) were included, as well as some relevant lessons from emergency settings.

### 2.2 Questionnaires and key informant interviews

Primary data came from key informants. Working group members developed questionnaires pertinent to the topics within their scope. The WG1 questionnaire covered topics related to policy, planning, and coordination of MNP programmes. The WG2 questionnaire covered topics related to delivery strategies, SBCC, and training for MNP programmes. The WG3 questionnaire covered topics related to monitoring, process evaluation, and supervisory systems for continual programme improvement. Key informants were identified through the consultation process using purposive and snowball sampling and interviewed by designated working group members, either in person or via telephone, using structured questionnaires. Some working group members also served as key informants for their own or other working groups. If key informants were not available for an interview, they were asked to complete a questionnaire. Verbal consent was obtained from key informants to be interviewed or to complete the questionnaire. The structured interviews guides/questionnaires were designed to garner experiences of programme implementers from a variety of country contexts. Informant interviews were analysed by working group members to identify common themes and programme examples. Where possible, authors attempted to triangulate personal opinions through published documents, follow‐up interviews, and in discussion with experts. The key informants were providing expert opinion as part of their professional capacity and regular public health practice. Thus, the activities involved in the consultation process were considered exempt by the John Snow, Inc. Institutional Review Board. Interview participants were told their names would be confidential in all reports and manuscripts and that any information gathered would be summarized in manuscripts submitted for peer‐review publication. Terms and general working definitions for the Consultation are presented in Box 1. The authors acknowledge that other definitions may apply outside the context of this paper. Each paper includes more specific definitions for their topic areas.

### 2.3 Literature search

We undertook a systematic literature search to identify papers with MNP programming relevance, to be used as secondary data in this programme review. We included articles using the following criteria: (a) MNP as an intervention provided to children 6–59 months of age, (b) relevant learning for MNP implementation, and (c) full text available in English. Exclusion criteria included: formulation or safety trials, registrations of clinical trials, press releases, commentaries, and editorials. We searched the following databases from inception to December 2015: PubMed, Conference Proceedings Citation Index—Science, EMBASE, Web of Science, New York Academy of Medicine's Grey Literature Database, Proquest Dissertation, and Theses Fulltext. For EMBASE and Web of Science, we restricted the publication type to technical report, dissertation, meeting paper, annual report, government publication, and programme report. We used a search strategy that combined various terms for MNP, which was modified from the search strategy in the Cochrane review on efficacy of MNP (De‐Regil et al., 2013). Supporting Information S2 provides details of the search engines and terms. The search results were imported into an Excel spreadsheet, where two reviewers independently screened the titles and abstracts and assessed their eligibility for inclusion in the review. The full texts of articles were obtained and reviewed when necessary, and all disagreements were resolved by consensus and, if required, a third review author. We also conducted a manual search for documents on the websites of 15 organizations who form the HF‐TAG, as well as the HF‐TAG website itself, using the same search terms. We reviewed all available literature, including powerpoints, technical reports, guidance, and other documents. During the consultation process, additional documents not found during the formal search were added by working group members. Finally, a list of known country‐level programmes was compiled from the 2011 Global Assessment (UNICEF & CDC, 2013) HF‐TAG website, NutriDash 2013 (UNICEF, 2014), and new programmes learned of through the consultation process. All resources were then made available to working groups for review during the consultative process.

## 3 RESULTS

Working groups reviewed the data from the systematic literature search, as well as 47 key informant interviews or questionnaires drawing on experiences working on MNP interventions in at least 35 countries, namely, Afghanistan, Bangladesh, Bolivia, Cambodia, Cameroon, China, Colombia, Dominican Republic, El Salvador, Ethiopia, Guatemala, India, Indonesia, Kenya, Kyrgyzstan, Lao PDR, Liberia, Madagascar, Mexico, Mongolia, Mozambique, Namibia, Nepal, Niger, Nigeria, Pakistan, Peru, the Philippines, Rwanda, South Sudan, South Africa, Tanzania, Uganda, Vietnam, and Zambia.

**Figure 2:**
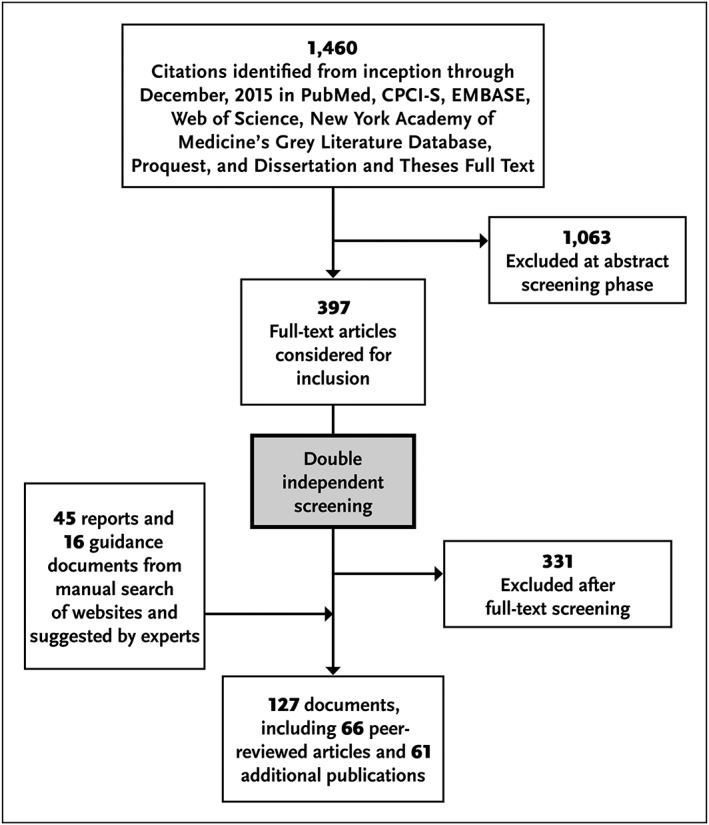
Literature search flow chart. The literature search flow chart presents the process undertaken to conduct to systematic review of literature related to micronutrient powders (MNP) programmes. Articles were included using the following criteria: (a) MNP as an intervention to children 6–59 months of age; (b) relevant learning for MNP intervention implementation; and (c) full text available in English. Exclusion criteria included formulation or safety trials, registrations of clinical trials, press releases, commentaries, and editorials. A systematic search was conducted of the following databases from inception of the Consultation to December 2015: PubMed, Conference Proceedings Citation Index—Science (CPCI‐S), EMBASE, Web of Science, New York Academy of Medicine's Grey Literature Database, Proquest Dissertation, and Theses Fulltext. A search strategy that combined various terms for MNP was modified from the search strategy used in the Cochrane review on efficacy of MNP (De‐Regil et al., 2013).

The systematic literature search identified 3,082 documents. After removal of duplicates, there were 1,460 unique abstracts. Of these, 1,063 were excluded in the abstract screening phase. The remaining 397 documents underwent duplicate independent screening resulting in 66 peer‐reviewed articles. The most common reasons for exclusion were efficacy studies with no programme‐specific learning or abstracts. To avoid double counting, we also removed dissertations that were later published. In addition, 45 reports, presentations, and other grey literature, and 16 global resource documents on implementation guidance were found resulting in 127 documents for review. A final list of identified literature is provided in Supporting Information S3. The flow diagram in Figure 2 identifies the articles included and excluded at various stages of the screening process.

### 3.1 Planning & Supply

The findings of the first working group (Schauer et al., 2017) centre around experiences during the planning phase whether at start‐up or scale‐up, of an MNP intervention, covering planning areas of assessment, enabling environment, adaptation, as well as supply considerations. Conducting high‐quality research and/or analyzing existing reliable data to justify an MNP intervention are considered a valuable first practice but have not always been conducted fully. Aside from local leadership and policy integration, the enabling environment for MNP at the country level has been aided by the need for a new approach to combat childhood anaemia. Funding MNP interventions remains a continuous challenge in most programmes, despite evidence of high cost effectiveness (Lopez Boo, Palloni, & Urzua, 2014; Sharieff, Horton, & Zlotkin, 2006). Key informants advised discussing long‐term funding at the planning stage for better sustainability, in lieu of building programs from piecemeal funding. Adding MNP interventions onto existing large‐scale programmes and financing through government rather than donors are seen to have improved funding security and sustainability (examples coming from Mexico and the Dominican Republic's social protection programmes). Securing reliable and regular supply is a common problem across many countries. Experiences from various countries cite MNP discoloration, odours, and other quality issues that have had a negative ripple effect on programme outcomes, particularly around acceptability of the product and the reputation of the distributors (Afsana, Haque, Sobhan, & Shahin, 2014; de Pee et al., 2013, 2008, 2007; Schauer & Zlotkin, 2003). To meet the complex specification and quality requirements of MNP and alleviate costs, most countries opt to procure MNP through global pre‐approved suppliers. However, challenges remain around loss of product due to lengthy procurement lead times, and inflexibility in package design (i.e., translations into local language).

### 3.2 Delivery, Social & Behavior Change Communication, and Training

The second working group present findings (Reerink et al., 2017) focusing on delivery, SBCC, and training. The authors review the available evidence on MNP delivery strategies based on different models (free, subsidized, or full‐cost product), platforms (e.g., health, social protection, and agriculture), and channels (e.g., facility health workers, community members, and pharmacists). Currently, the most common strategy to deliver MNP is as part of an infant and young child feeding programme, providing the product free through the health sector. Under this strategy, the highest coverage rates are seen where community distribution is employed. However, although this delivery strategy has shown advantages, funding and the added burden on struggling health systems remain a challenge. Providing MNP free through the nonhealth sector, such as social protection and early child development programmes, has also shown promise and has resulted in higher coverage rates compared to the health sector. Examples of distributing subsidized MNP are also discussed, but there has been substantial variation in the coverage of these programmes. Irrespective of the delivery strategy, MNP interventions are increasingly designed to establish links with broader infant and young child feeding objectives. Appropriate use and intake adherence, and not just coverage, are key behaviour outcomes on which programmes now measure their success (de Barros & Cardoso, 2016), though reliable and standardized metrics for these are limited. Implementers stressed the need for SBCC to be incorporated throughout the project life cycle in order to improve adoption of a set of minimal behaviours to achieve nutritional impact. In addition, regular refresher training of MNP distributors is seen as critical to ensure high‐quality counselling and messaging throughout programme stages and adaptations.

### 3.3 Continous Program Improvement

The last paper of the series (Vossenaar et al., 2017) presents the findings of the third working group on experiences related to MNP‐specific monitoring, process evaluation, and supportive supervision systems for continual programme improvement. The authors find that the ability to make evidence‐based decisions to improve MNP programme implementation is hindered by the lack of documented MNP experiences, particularly related to supportive supervision and among programmes implemented at scale. They identified 15 peer‐reviewed papers that investigate and describe factors (positive and negative) affecting MNP programme implementation, and have been used for programme adaptation. Most of these published experiences come from pilots. Although a common standard for effective programmes is the clear mapping of programme theory, most MNP programmes do not apply programme theories to track progress or make course corrections efficiently. They offer a case study to show how this process, in one context, has been aided by tools such as programme impact pathways and interactive learning agendas. Although monitoring systems require adequate allocation of financial and human resources, these are often lacking or inconsistent throughout the programme cycle. Furthermore, the insufficient capacity for monitoring MNP interventions is noted as a frequent challenge, particularly as pilot phases end and intensive technical assistance recedes. To be effective for programme improvement, they stress that monitoring systems require prioritization of the information to be collected, and explicit feedback loops for streamlined data compilation, interpretation, and utilization. A second case study presented illustrates how integration of MNP interventions into existing monitoring systems for scaled programmes is likely to require extensive planning and budgeting but is warranted to address programme quality. Another case study illustrates how ongoing process evaluations coupled with routine monitoring can be useful in triangulating and therefore verifying monitoring data, but authors note that correct utilization and sustaining of such parallel methods has rarely been prioritized within programme funding. They stress that the MNP implementation community remains tasked with documenting learning around programme sustainability and effectiveness.

## 4 DISCUSSION

Although the efficacy of MNP to reduce the risk of IDA has been well established, and extensive technical guidance and tools have been developed over the last two decades, implementing MNP interventions effectively remains a complex challenge. The Consultation and the resulting papers in this supplement identify some common elements across country programmes that facilitate or impede MNP implementation in the broad areas of planning, delivery, and programme improvement.

The papers in this supplement are based on MNP implementation literature and experts' experiences and learning; however, this consultative process had several methodological limitations. First, the state of the literature is such that most documents focused on efficacy and therefore programme learning was often not explicit. The lack of action‐oriented research in the area of nutrition is known (Pham & Pelletier, 2015). Second, although many countries and organizations were included in this process, the authors of these papers acknowledge that some countries' experiences may have been missed. Third, the review is not exhaustive and the information provided by key informants was based on personal experiences; therefore, the findings should be treated as expert opinion. As such, we do not intend to make broad scale inferences, and it should be recognized that other stakeholders involved in the implementation of MNP might have differing experiences or viewpoints. This type of summative process on programme experiences is still fairly methodologically new, and the use of expert opinion as the primary data source is subject to author interpretation.

Despite efficacy evidence and much advocacy for implementation of MNP—considered among the most cost‐effective interventions available to combat iron deficiency and anaemia in children (Bhutta et al., 2008)—funding constraints remain a challenge in almost all contexts. In particular, these papers highlight the importance of high‐quality MNP product, dynamic behaviour change communication, initial and ongoing training, capacity development, regular monitoring, and implementation research. It is therefore likely that the total cost of implementing sustainable MNP interventions is higher than previously estimated costs ($7.20 per child per year for supply and delivery, Horton et al., 2010), which do not encompass these emerging quality programming gaps. Updated costing studies would be very useful for realistic planning. Currently, two thirds of all MNP interventions are funded entirely by development partners (UNICEF, 2015); therefore, programmes aiming for long‐term sustainability may need to consider careful targeting of MNP interventions and/or using existing infrastructure and systems.

In addition, MNP interventions have had mixed success with changing behaviours (Reerink et al., 2017). This requires a shift in focus from just achieving high coverage to also considering programming issues including adherence and appropriate use (Neufeld, Piwoz, & Vasta, 2016). Aside from a behaviour‐centred approach, programming through more than one model, platform, or channel may be needed, depending on the context. Finally, weak monitoring systems may not be providing reliable data on reach (UNICEF, 2015) and rarely have built‐in mechanisms to inform regular programme improvement. More high‐quality process evaluations of MNP programmes are needed to describe the constraints and challenges of implementing and sustaining an effective programme, across varying delivery strategies. These would be most useful among ongoing programmes and assessed against an established theoretical programme impact pathway.

The lack of published or documented experience, particularly from larger or scaled MNP interventions, was noted across all working groups. As far as this review could establish, only four of the reported nine “national” MNP programmes (UNICEF, 2015; Schauer et al., 2017)—Bolivia, Brazil, Kyrgyzstan, and the Philippines—had any literature emerge in systematic search (and of these only two in peer‐reviewed publications). However, it should be noted that some of the mature large‐scale programmes have documented programme lessons (notably Bangladesh, Dominican Republic, Mexico, and Mongolia). Most existing documentation on MNP programme learning focuses on formative research and acceptability trials—learning usually generated early on and often only in pilot programmes. Although intensively examined pilots have their value for context‐specific implementation, systems‐based learning from large programmes (integration, national coordination, monitoring, supervision, sustainability, and supply) is better placed to inform sustainability and scale‐up. Bangladesh's MNP programme learning agenda, as described in Vossenaar et al. (2017), serves as an example of this systematic and continuous learning approach to programming. A clearly articulated global agenda for MNP programme learning would help prioritize future implementation research and programmes should budget for dissemination of programme learning so that other countries can learn from them.

The following list represents some areas of implementation research identified during the consultative process. They do not represent a prioritized or exhaustive list but are indicative of some the gaps in knowledge encountered while conducting this consultation:

- Develop basic formative research questions, methodologies, and tools that most programmes can use to inform the programming of and communication around MNP focusing on the context‐specific knowledge, attitudes, and behaviours around complementary feeding and other aspects that might impact adoption;
- Identify the “tipping points” to achieving government buy‐in (political and financial) of MNP and related contextual factors;
- Articulate decision‐making pathways to source MNP according to capacity requirements and regulatory and import tariff regimes;
- Determine if a mixed or subsidized model can maintain equitable accessibility to those at risk of iron deficiency or anaemia while remaining viable and sustainable (i.e., with acceptable profit margins to keep the private‐sector engaged);
- Determine how to manage the burden of adding MNP delivery to frontline staff workload, as well as testing different types of incentives to retain and motivate delivery channel distributors;
- Define common indicators and metrics for programme performance that are linked to nutritional impact;
- Examine how to effectively link monitoring and process evaluation to decision‐making processes;
- Document lessons in how to sustain monitoring systems from pilot to larger scale;
- Document how to carry out effective supportive supervision, especially in contexts with high turnover of MNP staff.

Infant and young child anaemia caused by iron deficiency is a pervasive problem with few efficacious interventions. Successful implementation of evidence‐based interventions, such as MNP, is required to address this long‐standing challenge. In controlled contexts, a promising 34% reduction in anaemia can be achieved through MNP (De‐Regil et al., 2013). Programme learning should continue to be a part of MNP implementation using rigorous process evaluations. This information will contribute to the high‐quality and large‐scale implementation of MNP interventions and build the efficiency of this high‐potential intervention.

## CONFLICT OF INTEREST

The authors declare no conflicts of interest.

## CONTRIBUTIONS

This paper was written by CND, DS, and SN and reviewed by RK, LMN, RR, and AT. All authors were involved in developing the paper concept and have reviewed and approved the submitted manuscript.

## Supporting information

Supporting Information S1. Supplementary Material 1. List of Consultation Participants^1^

Supporting Information S2. Supplementary Material 2. Literature Review Search engines and terms

Supporting Information S3. Supplementary Material 3. Literature Search Results
