# Supplementary material for: Executive summary for the Micronutrient Powders Consultation: Lessons Learned for Operational Guidance
Source: Matern Child Nutr. 2017 Sep 29;13(Suppl 1):e12493. doi: 10.1111/mcn.12493 (PMC5656884; doi:10.1111/mcn.12493)
Supplement: Supplementary file 1 — Supporting Information S1. Supplementary Material 1. List of Consultation Participants1 [file MCN-13-e12493-s001.docx]

**Supplementary Material 1: List of Consultation Participants**^1^

| **Full Name** | **Affiliation & Country** |
| --- | --- |
| Canahuati, Judy | USAID, USA |
| DeBernardo, Diane | USAID, USA |
| Hubbell Melgarejo, Carrie (Rapporteur) | SPRING, USA |
| Klemm, Rolf (Chair) | Helen Keller International, USA |
| Walton, Shelley | Tufts University, USA |
| Possolo, Edna | Government of Mozambique |
| Quick, Timothy | USAID, USA |
| Roca, Claudia | DELIVER PROJECT, JSI Guatemala |
| Schauer, Claudia | The Hospital for Sick Children, Canada |
| Situma, Ruth | UNICEF, USA |
| Sunley, Nigel | Sunley Consulting, South Africa |
| Tapia Terán, Gustavo Iván | Independent Consultant, Bolivia |
| Zlotkin, Stanley | The Hospital for Sick Children, Canada |
| Abbott, Sally | USAID, USA |
| Aburto, Nancy | World Food Programme, Italy |
| Bontrager, Elizabeth | USAID, USA |
| Chaudhery, Deepika Nayar | Micronutrient Initiative, India |
| Griffiths, Marcia | Manoff Group, USA |
| Haque, Mohammad Raisul | BRAC, Bangladesh |
| Jefferds, Maria Elena | CDC/IMMPaCT, USA |
| Kroeun, Hou | Helen Keller International, Cambodia |
| Namaste, Sorrel (Rapporteur) | SPRING, USA |
| Rawat, Rahul (Chair) | International Food Policy Research Institute, Senegal |
| Reerink, Ietje | Independent, Madagascar |
| Bonvecchio Arenas, Anabelle | National Institute of Public Health,  Mexico |
| D'Agostino, Alexis (Rapporteur) | SPRING, USA |
| Grajeda, Ruben | PAHO/WHO, USA |
| Gray, Elaine | USAID, USA |
| Imanalieva, Cholpon | UNICEF, Kyrgyzstan |
| Irizarry, Laura | Independent Consultant, Peru |
| Maalouf-Manasseh, Zeina | FHI360, USA |
| Mulokozi, Generose | Government of Tanzania |
| Neufeld, Lynnette (Co-chair) | Global Alliance for Improved Nutrition, Switzerland |
| Noto Sudardjo, Minarto | Millennium Challenge Account, Indonesia |
| Thurber, Melanie | USAID, USA |
| Tsevegsuren, Narantsetseg | World Vision International, Mongolia |
| Tumilowicz, Alison (Co-chair) | Global Alliance for Improved Nutrition, Switzerland |
| Zimmermann, Michael | Federal Institute of Technology, Switzerland |
| Bahl, Kanika | Results For Development, USA |
| Cotes, Gwyneth | SPRING, USA |
| Dary, Omar | USAID, USA |
| Makonnen, Raphael | USAID, USA |
| Mouw, Traci | USDA, USA |
| Nyhus Dhillon, Christina (Facilitator) | SPRING Consultant, USA |
| Rosenberg, Irwin | Tufts University, USA |
| Sarkar, Danya | SPRING, USA |
| Schaan, Michelle | USAID, USA |
| Stewart, Kellie | USAID, USA |
| Wetzel-Chen, Carolyn | Millennium Challenge Account, USA |

1-USAID, United States Agency for International Development; SPRING, Strengthening Partnerships, Results, and Innovations in Nutrition Globally; JSI, John Snow Institute; UNICEF, United Nations Children’s Fund; BRAC, Building Resources Across Communities; CDC, Centers for Disease Control and Prevention; IMMPaCT, International Micronutrient Malnutrition Prevention and Control; PAHO, Pan American Health Organization; WHO, World Health Organization; FHI360, Family Health International 360; USDA, United States Department of Agriculture.
