# Supplementary material for: Executive summary for the Micronutrient Powders Consultation: Lessons Learned for Operational Guidance
Source: Matern Child Nutr. 2017 Sep 29;13(Suppl 1):e12493. doi: 10.1111/mcn.12493 (PMC5656884; doi:10.1111/mcn.12493)
Supplement: Supplementary file 2 — Supporting Information S2. Supplementary Material 2. Literature Review Search engines and terms [file MCN-13-e12493-s002.docx]

**Supplementary Material 2:** **Literature Review Search engines and terms**

1. Search engines:

PubMed

MEDLINE

Conference Proceedings Citation Index - Science (CPCI-S)

EMBASE (limit publication type to technical report, dissertation, meeting paper, annual report, government publication, program report^[[1]](#footnote-1)^)

Web of Science (limit publication type to technical report, dissertation, meeting paper, annual report, government publication, program report)

New York Academy of Medicine- Grey Literature Database

Proquest Dissertation and Theses Fulltext (formerly Dissertation Abstracts)

1. On the following search terms^[[2]](#footnote-2)^:

# 1 [micronutrient* or micro-nutrient* or micro next nutrient* or multimicronutrient* or multimicro next nutrient*] AND [mix$ or powder$ or supplement$ or sachet$ or packet$ or powder$ or MNP or MNPs]

# 2 “Point of use” AND “fortification” (adding fortification deviates from Regil)

#3 “Home fortification”

#4 Sprinkles or Vita Shakti or Rahama or Anuka or Chispitas or BabyFer or Bebe Vanyan or Supplefer or Supplefem

1. As suggested by UPenn Library Grey Literature Search Strategies (<http://guides.library.upenn.edu/healthgreylit>) [↑](#footnote-ref-1)
2. Search terms taken from Cochrane Review: Home fortiﬁcation of foods with multiple micronutrient powders for health and nutrition in children under two years of age (Review) Copyright © 2011 The Cochrane Collaboration. Published by John Wiley & Sons, Ltd. [↑](#footnote-ref-2)
