# Supplementary material for: Executive summary for the Micronutrient Powders Consultation: Lessons Learned for Operational Guidance
Source: Matern Child Nutr. 2017 Sep 29;13(Suppl 1):e12493. doi: 10.1111/mcn.12493 (PMC5656884; doi:10.1111/mcn.12493)
Supplement: Supplementary file 3 — Supporting Information S3. Supplementary Material 3. Literature Search Results [file MCN-13-e12493-s003.doc]

**Supplementary Material 3: Literature Search Results**

**Peer-Reviewed Journal Articles, by author**

| **No** | **Author(s)** | **Year** | **Title** | **Journal/Book Title** |
| --- | --- | --- | --- | --- |
| **1** | Adrianopoli, M; D'Acapito, P; Ferrari, M; Mistura, L; Toti, E; Maiani, G; Truebswasser, U; Boymatova, K; Severoni, S | 2014 | Optimized feeding recommendations and in-home fortification to improve iron status in infants and young children in the Republic of Tajikistan: A pilot project | Improving Diets and Nutrition: Food-Based Approaches |
| **2** | Adu-Afarwuah, S; Lartey, A; Brown, KH; Zlotkin, S; Briend, A; Dewey, KG | 2008 | Home fortification of complementary foods with micronutrient supplements is well accepted and has positive effects on infant iron status in Ghana | American Journal of Clinical Nutrition |
| **3** | Afsana, K; Haque, MR; Sobhan, S; Shahin, SA | 2014 | BRAC's experience in scaling-up MNP in Bangladesh | Asia Pacific Journal of Clinical Nutrition |
| **4** | Angdembe, MR; Choudhury, N; Haque, MR; Ahmed, T | 2015 | Adherence to multiple micronutrient powder among young children in rural Bangladesh: A cross-sectional study | BMC Public Health |
| **5** | Attanasio, OP; Fernandez, C; Fitzsimons, EO; Grantham-McGregor, SM; Meghir, C; Rubio-Codina, M | 2014 | Using the infrastructure of a conditional cash transfer program to deliver a scalable integrated early child development program in Colombia: Cluster randomized controlled trial | BMJ |
| **6** | Avula, R; Frongillo, EA; Arabi, M; Sharma, S; Schultink, W | 2011 | Enhancements to nutrition program in Indian integrated Child Development Services increased growth and energy intake of children | Journal of Nutrition |
| **7** | Bilukha, O; Howard, C; Wilkinson, C; Bamrah, S; Husain, F | 2011 | Effects of multi-micronutrient home fortification on anemia and growth in Bhutanese refugee children | Food and Nutrition Bulletin |
| **8** | Boo, FL; Palloni, G; Urzua, S | 2014 | Cost-benefit analysis of a micronutrient supplementation and early childhood stimulation program in Nicaragua | Annals of the New York Academy of Sciences |
| **9** | Christofides, A; Schauer, C; Sharieff, W; Zlotkin, SH | 2005 | Acceptability of micronutrient sprinkles: A new food-based approach for delivering iron to First Nations and Inuit children in Northern Canada | Chronic Diseases in Canada |
| **10** | Christofides, A; Schauer, C; Zlotkin, SH | 2005 | Iron deficiency anemia among children: Addressing a global public health problem within a Canadian context | Paediatrics and Child Health |
| **11** | Creed-Kanashiro, H; Bartolini, R; Abad, M; Arevalo, V | 2015 | Promoting multi-micronutrient powders (MNP) in Peru: acceptance by caregivers and role of health personnel | Maternal & Child Nutrition |
| **12** | de Barros, SF; Cardosa, MA | 2016 | Adherence to and acceptability of home fortification with vitamins and minerals in children aged 6 to 23 months: a systematic review | BMC Public Health |
| **13** | de Pee, S; Kraemer, K; van den Briel, T; Boy, E; Grasset, C; Moench-Pfanner, R; Zlotkin, S; Bloem, MW | 2008 | Quality criteria for micronutrient powder products: Report of a meeting organized by the World Food Programme and Sprinkles Global Health Initiative | Food and Nutrition Bulletin |
| **14** | de Pee, S; Moench-Pfanner, R; Martini, E; Zlotkin, SH; Darnton-Hill, I; Bloem, MW | 2007 | Home fortification in emergency response and transition programming: Experiences in Aceh and Nias, Indonesia | Food and Nutrition Bulletin |
| **15** | de Pee, S; Spiegel, P; Kraemer, K; Wilkinson, C; Bilukha, O; Seal, A; Macias, K; Oman, A; Fall, AB; Yip, R; Pena-Rosas, JP; West, K; Zlotkin, S; Bloem, MW | 2011 | Assessing the impact of micronutrient intervention programs implemented under special circumstances: Meeting report | Food and Nutrition Bulletin |
| **16** | De-Regil, LM; Suchdev, PS; Vist, GE; Walleser, S; Pena-Rosas, JP | 2013 | Home fortification of foods with multiple micronutrient powders for health and nutrition in children under two years of age | Evidence-Based Child Health |
| **17** | Dewey, KG; Yang, Z; Boy, E | 2009 | Systematic review and meta-analysis of home fortification of complementary foods | Maternal and Child Nutrition |
| **18** | Fernandez-Rao, S; Hurley, KM; Nair, KM; Balakrishna, N; Radhakrishna, KV; Ravinder, P; Tilton, N; Harding, KB; Reinhart, GA; Black, MM | 2014 | Integrating nutrition and early child-development interventions among infants and preschoolers in rural India | Annals of the New York Academy of Sciences |
| **19** | Geltman, PL; Hironaka, LK; Mehta, SD; Padilla, P; Rodrigues, P; Meyers, AF; Bauchner, H | 2009 | Iron supplementation of low-income infants: A randomized clinical trial of adherence with ferrous fumarate sprinkles versus ferrous sulfate drops | Journal of Pediatrics |
| **20** | Harris, JL; Patelm MK; Juliao, P; Suchdev, PS; Ruth, JL; Were, V; Ochieng, C; Faith, SH; Kola, S; Otieno,R; Sadumah, I; Obure, A; Quick, R | 2012 | Addressing Inequities in Access to Health Products through the Use of Social Marketing, Community Mobilization, and Local Entrepreneurs in Rural Western Kenya | International Journal of Population Research |
| **21** | Hirve, S; Bhave, S; Bavdekar, A; Naik, S; Pandit, A; Schauer, C; Christofides, A; Hyder, Z; Zlotkin, S | 2007 | Low dose 'Sprinkles' - An innovative approach to treat iron deficiency anemia in infants and young children | Indian Pediatrics |
| **22** | Hirve, S; Martini, E; Juvekar, SK; Agarwal, D; Bavdekar, A; Sari, M; Molwane, M; Janes, S; Haselow, N; Yeung, DL; Pandit, A | 2013 | Delivering Sprinkles Plus through the Integrated Child Development Services (ICDS) to reduce anemia in pre-school children in India | Indian Journal of Pediatrics |
| **23** | Hou, J; Sun, J; Fang, Z; Chang, S; Zhao, L; Fu, P; Wang, J; Huang, J; Wang, L; Begin, F; Hipgrave, DB; Ma, G | 2015 | Effect of home-based complementary food fortification on prevalence of anemia among infants and young children aged 6 to 23 months in poor rural regions of China | Food and Nutrition Bulletin |
| **24** | Hyder, SMZ; Haseen, F; Rahman, M; Tondeur, MC; Zlotkin, SH | 2007 | Effect of daily versus once-weekly home fortification with micronutrient Sprinkles on hemoglobin and iron status among young children in rural Bangladesh | Food and Nutrition Bulletin |
| **25** | Inayati, DA; Scherbaum, V; Purwestri, RC; Wirawan, NN; Suryantan, J; Hartono, S; Bloem, MA; Pangaribuan, RV; Biesalski, HK; Hoffmann, V; Bellows, AC | 2012 | Combined intensive nutrition education and micronutrient powder supplementation improved nutritional status of mildly wasted children on Nias Island, Indonesia | Asia Pacific Journal of Clinical Nutrition |
| **26** | Ip, H; Hyder, SMZ; Haseen, F; Rahman, M; Zlotkin, SH | 2009 | Improved adherence and anaemia cure rates with flexible administration of micronutrient Sprinkles: A new public health approach to anaemia control | European Journal of Clinical Nutrition |
| **27** | Jack, SJ; Ou, K; Chea, M; Chhin, L; Devenish, R; Dunbar, M; Eang, C; Hou, K; Ly, S; Khin, M; Prak, S; Reach, R; Talukder, A; Tokmoh, L-O; Leon, De La Barra S; Hill, PC; Herbison, P; Gibson, RS | 2012 | Effect of micronutrient sprinkles on reducing anemia: A cluster-randomized effectiveness trial | Archives of Pediatrics and Adolescent Medicine |
| **28** | Jefferds ME; Irizarry L; Timmer A; Tripp K | 2013 | UNICEF-CDC global assessment of home fortification interventions 2011: Current status, new directions, and implications for policy and programmatic guidance | Food and Nutrition Bulletin |
| **29** | Jefferds, ME; Flores-Ayala, R | 2015 | Introducing a new monitoring manual for home fortification and strengthening capacity to monitor nutrition interventions | Maternal and Child Nutrition |
| **30** | Jefferds, ME; Mirkovic, KR; Subedi, GR; Mebrahtu, S; Dahal, P; Perrine, CG | 2015 | Predictors of micronutrient powder sachet coverage in Nepal | Maternal and Child Nutrition |
| **31** | Jefferds, ME; Ogange, L; Owuor, M; Cruz, K; Person, B; Obure, A; Suchdev, PS; Ruth, LJ | 2010 | Formative research exploring acceptability, utilization, and promotion in order to develop a micronutrient powder (Sprinkles) intervention among Luo families in western Kenya | Food and Nutrition Bulletin |
| **32** | Kemmer, TM; Omer, PS; Gidvani-Diaz, VK; Coello, M | 2012 | Acceptance and effect of ferrous fumarate containing micronutrient Sprinkles on anemia, iron deficiency, and anthropometrics in Honduran children | InTech |
| **33** | Kodish, S; Rah, JH; Kraemer, K; De Pee, S; Gittelsohn, J | 2011 | Understanding low usage of micronutrient powder in the Kakuma refugee camp, Kenya: Findings from a qualitative study | Food and Nutrition Bulletin |
| **34** | Kounnavong, S; Sunahara, T; Mascie-Taylor, CGN; Hashizume, M; Okumura, J; Moji, K; Boupha, B; Yamamoto, T | 2011 | Effect of daily versus weekly home fortification with multiple micronutrient powder on haemoglobin concentration of young children in a rural area, Lao People's Democratic Republic: A randomised trial | Nutrition Journal |
| **35** | Loechl, CU; Menon, P; Arimond, M; Ruel, MT; Pelto, G; Habicht, J-P; Michaud, L | 2009 | Using programme theory to assess the feasibility of delivering micronutrient Sprinkles through a food-assisted maternal and child health and nutrition programme in rural Haiti | Maternal and Child Nutrition |
| **36** | Lundeen, E; Schueth, T; Toktobaev, N; Zlotkin, S; Hyder, SMZ; Houser, R | 2010 | Daily use of Sprinkles micronutrient powder for 2 months reduces anemia among children 6 to 36 months of age in the Kyrgyz Republic: A cluster-randomized trial | Food and Nutrition Bulletin |
| **37** | Menon, P; Rawat, R; Saha, KK; Roopnaraine, T; Khaled, A; Bhuiyan, MI; Islam, MA; Khan, MA; Siraj, S; Ruel, MT | 2013 | A mixed-methods process evaluation (PE) using program impact pathway (PIP) highlights the role of demand creation in implementing a market-based micronutrient powder (MNP) intervention in rural Bangladesh | FASEB Journal |
| **38** | Menon, P; Ruel, MT; Loechl, CU; Arimond, M; Habicht, J-P; Pelto, G; Michaud, L | 2007 | Micronutrient Sprinkles reduce anemia among 9- to 24-mo-old children when delivered through an integrated health and nutrition program in rural Haiti | Journal of Nutrition |
| **39** | Mirkovic, KR; Perrine, CG; Subedi, GR; Mebrahtu, S; Dahal, P; Staatz, C; Jefferds, MED | 2015 | Predictors of micronutrient powder intake adherence in a pilot programme in Nepal | Public Health Nutrition |
| **40** | Ndemwa, P; Klotz, CL; Mwaniki, D; Sun, K; Muniu, E; Andango, P; Owigar, J; Rah, JH; Kraemer, K; Spiegel, PB; Bloem, MW; de Pee, S; Semba RD | 2011 | Relationship of the availability of micronutrient powder with iron status and hemoglobin among women and children in the Kakuma Refugee Camp, Kenya | Food and Nutrition Bulletin |
| **41** | Nguyen, M; Poonawala, A; Leyvraz, M; Berger, J;Schofield, D; Nga, TT; Van, TK; Hoa, DTB; Wieringa, FT | 2016 | A Delivery Model for Home Fortification of Complementary Foods with Micronutrient Powders: Innovation in the Context of Vietnamese Health System Strengthening | Nutrients |
| **42** | Ogunlade, AO; Kruger, HS; Jerling, JC; Smuts, CM; Covic, N; Hanekom, SM; Mamabolo, RL; Kvalsvig, J | 2011 | Point-of-use micronutrient fortification: Lessons learned in implementing a preschool-based pilot trial in South Africa | International Journal of Food Sciences and Nutrition |
| **43** | Osei, A; Septiari, A; Suryantan, J; Hossain, MdM; Chiwile, F; Sari, M; Pinto, P; Soares, D; Faillace, S | 2014 | Using formative research to inform the design of a home fortification with micronutrient powders (MNP) program in Aileu District, Timor-Leste | Food and Nutrition Bulletin |
| **44** | Osei, AK; Pandey, P; Spiro, D; Adhikari, D; Haselow, N; De Morais, C; Davis, D | 2015 | Adding multiple micronutrient powders to a homestead food production programme yields marginally significant benefit on anaemia reduction among young children in Nepal | Maternal and Child Nutrition |
| **45** | Pelto, GH; Armar-Klemesu, M; Siekmann, J; Schofield, D | 2013 | The focused ethnographic study 'assessing the behavioral and local market environment for improving the diets of infants and young children 6 to 23 months old' and its use in three countries | Maternal and Child Nutrition |
| **46** | Rah, JH; De Pee, S; Halati, S; Parveen, M; Mehjabeen, SS; Steiger, G; Bloem, MW; Kraemer, K | 2011 | Provision of micronutrient powder in response to the cyclone Sidr emergency in Bangladesh: Cross-sectional assessment at the end of the intervention | Food and Nutrition Bulletin |
| **47** | Rah, JH; dePee, S; Kraemer, K; Steiger, G; Bloem, MW; Spiegel, P; Wilkinson, C; Bilukha, O | 2012 | Program experience with micronutrient powders and current evidence | Journal of Nutrition |
| **48** | Rezaul, K; Desplats, G; Schaetzel, T; Herforth, A; Ahmed, F; Salamatullah, Q; Shahjahan, M; Akhtaruzzaman, M; Levinson, J | 2005 | Seeking optimal means to address micronutrient deficiencies in food supplements: A case study from the Bangladesh Integrated Nutrition Project | Journal of Health, Population, and Nutrition |
| **49** | Salam, RA; MacPhail, C; Das, JK; Bhutta, ZA | 2013 | Effectiveness of micronutrient powders (MNP) in women and children | BMC Public Health |
| **50** | Sarwar, MR | 2015 | Bangladesh health service delivery: Innovative NGO and private sector partnerships | IDS Bulletin |
| **51** | Sazawal, S; Dhingra, P; Dhingra, U; Gupta, S; Iyengar, V; Menon, VP; Sarkar, A; Black, RE | 2014 | Compliance with home-based fortification strategies for delivery of iron and zinc: Its effect on haematological and growth markers among 6-24 months old children in North India | Journal of Health, Population, and Nutrition |
| **52** | Serdula, MK; Lundeen, E; Nichols, EK; Imanalieva, C; Minbaev, M; Mamyrbaeva, T; Timmer, A; Aburto, NJ; Samohleb, G; Donnie, WR; Mandava, U; Sullivan, KM | 2013 | Effects of a large-scale micronutrient powder and young child feeding education program on the micronutrient status of children 6-24 months of age in the Kyrgyz Republic | European Journal of Clinical Nutrition |
| **53** | Sharieff, W; Horton, SE; Zlotkin, S | 2006 | Economic gains of a home fortification program: Evaluation of "Sprinkles" from the provider's perspective | Canadian Journal of Public Health |
| **54** | Sharieff, W; Yin, S-A; Wu, M; Yang, Q; Schauer, C; Tomlinson, G; Zlotkin, S | 2006 | Short-term daily or weekly administration of micronutrient Sprinkles™ has high compliance and does not cause iron overload in Chinese schoolchildren: A cluster-randomised trial | Public Health Nutrition |
| **55** | Sharieff, W; Zlotkin, SH; Ungar, WJ; Feldman, B; Krahn, MD; Tomlinson, G | 2008 | Economics of preventing premature mortality and impaired cognitive development in children through home-fortification: A health policy perspective | International Journal of Technology Assessment in Health Care |
| **56** | Sharieff, W; Zlotkin, S; Feldman, B; Tomlinson, G; Ungar, W; Krahn, M | 2004 | Quantifying economic gains of supplementing Pakistani infants living in urban slums with micronutrient 'Sprinkles': A cost-benefit analysis using computer simulation techniques | FASEB Journal |
| **57** | Style, S; Tondeur, M; Wilkinson, C; Oman, A; Spiegel, P; Kassim, IAR; Grijalva-Eternod, C; Dolan, C; Seal, A | 2013 | Operational guidance on the use of special nutritional products in refugee populations | Food and Nutrition Bulletin |
| **58** | Suchdev, PS; Ruth, L; Obure, A; Were, V; Ochieng, C; Ogange, L; Owuor, M; Ngure, F; Quick, R; Juliao, P; Jung, C; Teates, K; Cruz, K; Jefferds, MED | 2010 | Monitoring the marketing, distribution, and use of Sprinkles micronutrient powders in rural western Kenya | Food and Nutrition Bulletin |
| **59** | Suchdev, PS; Ruth, LJ; Woodruff, BA; Mbakaya, C; Mandava, U; Flores-Ayala, R; Jefferds, MED; Quick, R | 2012 | Selling Sprinkles micronutrient powder reduces anemia, iron deficiency, and vitamin A deficiency in young children in Western Kenya: A cluster-randomized controlled trial | American Journal of Clinical Nutrition |
| **60** | Suchdev, PS; Shah, A; Jefferds, MED; Eleveld, A; Patel, M; Stein, AD; MacDonald, B; Ruth, L | 2013 | Sustainability of market-based community distribution of Sprinkles in Western Kenya | Maternal and Child Nutrition |
| **61** | Sun, J; Dai, YH; Zhang, SM; Huang, J; Yang, ZY; Huo, JS; Chen, CM | 2011 | Implementation of a programme to market a complementary food supplement (Ying Yang Bao) and impacts on anaemia and feeding practices in Shanxi, China | Maternal and Child Nutrition |
| **62** | Tran, VK; Spohrer, R; Le, TD; Poonawala, A; Monech-Pfanner, R | 2015 | Micronutrient deficiency control in Vietnam from policy and research to implementation: Keys for success, challenges, and lessons learned | Journal of Nutritional Science and Vitaminology |
| **63** | Tripp, K; Perrine, CG; de Campos, P; Knieriemen, M; Hartz, R; Ali, F; Jefferds, MED; Kupka, R | 2011 | Formative research for the development of a market-based home fortification programme for young children in Niger | Maternal and Child Nutrition |
| **64** | Vijay, J; Sharma, S | 2015 | Impact of micronutrient Sprinkles on weight and height of children aged 6-36 months in Tonk district of Rajasthan State | Indian Journal of Community Health |
| **65** | Young, SL; Blanco, I; Hernandez-Cordero, S; Pelto, GH; Neufeld, LM | 2010 | Organoleptic properties, ease of use, and perceived health effects are determinants of acceptability of micronutrient supplements among poor Mexican women | Journal of Nutrition |
| **66** | Zlotkin, S; Siekmann, J; Lartey, A; Yang, Z | 2010 | The role of the Codex Alimentarius process in support of new products to enhance the nutritional health of infants and young children | Food and Nutrition Bulletin |

**Grey Literature, by organization**

| **No** | **Organization(s)/ Author(s)** | **Year** | **Title** | **Format** |
| --- | --- | --- | --- | --- |
| **1** | Alive & Thrive / Dewey, K; Vitta, BS | 2013 | Strategies for ensuring adequate nutrient intake for infants and young children during the period of complementary feeding | Technical Report |
| **2** | Armstrong, AL | 2009 | Anemia in Central-Asia pre-school children: Definition, risk factors and evaluation of home fortification intervention | Dissertation |
| **3** | Business Innovation Facility | 2013 | Commercial home fortification projects: Bangladesh political economy mapping | Report |
| **4** | *Estudo Nacional de Fortificação caseira da Alimentação Complementar* (ENFAC) | Unknown | Report on Brazilian study for home fortification of complementary feeding | Report |
| **5** | Food and Nutrition Technical Assistance II / Mridha, MK; Chaparro, CM; Matias, SL; Hussain, S; Munira, S; Saha, S; Day, LT; Dewey, KG | 2012 | Acceptability of lipid-based nutrient supplements and micronutrient powders among pregnant and lactating women and infants and young children in Bangladesh and their perceptions about malnutrition and nutrient supplements | Technical Report |
| **6** | Food and Nutrition Technical Assistance Project | 2014 | Development of evidence-based dietary recommendations for children, pregnant women, and lactating women living in the Western Highlands in Guatemala | Technical Report |
| **7** | Food and Nutrition Technical Assistance Project III / Olney, D; Arriola, M; Carranza, R; Leroy, J; Richter, S; Harris, J; Ruel, M; Becker, E | 2012 | Report of formative research conducted in Alta Verapaz, Guatemala, to help inform the health-strengthening activities and the social and behavior change communication strategy that will be implemented through the Mercy Corps PM2A Program – PROCOMIDA | Technical Report |
| **8** | Food and Nutrition Technical Assistance Project III / Olney, D; Richter, S; Becker, E; Roopnaraine, T; Margolies, A; Kennedy, A; Leroy, J; Ruel, M | 2013 | A process evaluation of the PROCOMIDA “Preventing malnutrition in children under 2 approach” in Guatemala | Technical Report |
| **9** | Global Alliance for Improved Nutrition | 2015 | Infant & Young Child Feeding in Cambodia, Vietnam and Laos: Workshops to share lessons learned and good practices in home fortification of complementary foods with micronutrient powders meeting report | Meeting Report |
| **10** | Global Alliance for Improved Nutrition | 2015 | Infant & Young Child Feeding in South Africa meeting report | Meeting Report |
| **11** | Global Alliance for Improved Nutrition | 2012 | A child's daily nutrition within a small sachet | Case Study |
| **12** | Global Alliance for Improved Nutrition / van Liere, MJ; Frega, R; Tarlton, D; Schofield, D | 2015 | Improving complimentary feeding: Assessing public and private sector business models (IYCN Paper 1) | Technical Paper |
| **13** | Global Alliance for Improved Nutrition / van Liere, MJ; Godfrey, S; Siekmann, J; Badham, J; Schofield, D | 2016 | Strengthening the enabling environment for scaling-up access to good quality complementary foods (IYCN Paper 3) | Technical Paper |
| **14** | Global Alliance for Improved Nutrition / van Liere, MJ; Poonawala, A | 2015 | Promoting optimal infant feeding practices and effective use of complementary foods for infants: Delivery lessons (IYCN Paper 2) | Technical Paper |
| **15** | Helen Keller International & Irish Aid | 2015 | Enhancing coverage of and adherence to in-home fortification of micronutrient powders | Project Final Report |
| **16** | Home Fortification Technical Advisory Group | 2011 | Global assessment Report 2011 | Technical Report |
| **17** | Hospital for Sick Children Canada, Micronutrient Initiative, Universidad Mayor de San Andres / Schauer, C; Harding, K; MacLean, A; Vaccarino, O; Roche, ML; Aguilar, AM; Zlotkin, S | 2014 | National scale up of micronutrient powders in Bolivia – a case study | Case Study |
| **18** | Manoff Group / Griffiths, M | 2011 | Improved complementary feeding practices with MNP: Blending two parallel programs into one | Powerpoint slides |
| **19** | Mathematica / Beatty, A; Borkum, E; Rangarajan, A; Gage, A; Null, C; Sethi, S | 2014 | MCC Indonesia nutrition project impact evaluation design September 12, 2014 | Technical Report |
| **20** | Micronutrient Initiative / MacLean, A | 2011 | National distribution of MNP in Bolivia | Powerpoint slides |
| **21** | Philippines Nutrition Cluster | 2014 | Overview of micronutrient powder (MNPs) / Vita Mix (VM) | Powerpoint slides |
| **22** | Population Services International / Reerink, I | 2015 | Social marketing of "Super Kid" micronutrient powder in Lao PDR | Powerpoint slides |
| **23** | Results for Development / Bahl, K; Jayaram, S; Brown, B | 2014 | DSM-WFP: A partnership to advance the global nutrition agenda | Technical Report |
| **24** | Results for Development / Bahl, K; Toro, E; Qureshi, C; Shaw, P | 2013 | Nutrition for a better tomorrow: Scaling up delivery of micronutrient powders for infants and young children | Technical Report |
| **25** | Sight and Life / Asis, R; van Hees, J; de Pee, S | 2013 | Home fortification in emergency situations: How introducing MNP in the Philippines in 2009 guided provision of MNP in development settings and the design of the national supplementation program. | Magazine |
| **26** | Sight and Life / de Pee, S; Irizarry, L; Kraemer, K; Jefferds, ME | 2013 | The basis for current programming guidance and needs for additional knowledge and experience. | Magazine |
| **27** | Sight and Life / Gittelsohn, J; Cristello, A | 2014 | Sustaining a national MNP supplementation program: Findings of the qualitative evaluation of the FORTIDOM pilot trail in Madagascar. | Magazine |
| **28** | Sight and Life / Halati, S; van Hees, J; Uprety, S; Schapendonk, E; de Pee, S | 2013 | Micronutrient powder supplementation program for Bhutanese refugee children in Jhapa and Morang Districts, Nepal. | Magazine |
| **29** | Sight and Life / Lundeen, E; Imanalieva, C; Mamyrbaeva, T; Timmer, A | 2013 | Integrating micronutrient powder into a broader child health and nutrition program in Kyrgyzstan | Magazine |
| **30** | Sight and Life / MacLean, A; Jalal, C; Loayza, M; Neufeld, LM | 2013 | Chispitas in Bolivia: Experience and case study. | Magazine |
| **31** | Sight and Life / Martini, E; van Hees, J; de Pee, S | 2013 | Distributing a micronutrient powder to tsunami-affected children in Indonesia. | Magazine |
| **32** | Sight and Life / Michaux, K; Anema, A; Green, T; Smith, L; McLean, J; Omwega, A; Ngabo, F; Brunet, D; Aongola, A; Lezama, I; Berdaga, V; Chiwileh, F; Ahimbisibwei, M | 2014 | Home fortification with micronutrient powders: Lessons learned from formative research across six countries. | Magazine |
| **33** | Sight and Life / Siekmann, J; Timmer, A; Irizarry, L | 2013 | The potential of market-based approaches to complement free public distribution of micronutrient powders. | Magazine |
| **34** | Sight and Life / Vanchinkhuu, S; Norov, B; Bat, G | 2013 | Introducing a micronutrient powder program in Mongolia. | Magazine |
| **35** | United Nations International Children's Emergency Fund | 2013 | NutriDash 2013 | Annual Report |
| **36** | United Nations International Children's Emergency Fund | 2015 | NutriDash 2014 | Annual Report |
| **37** | United Nations International Children's Emergency Fund | 2014 | Multiple micronutrient powder supply & outlook | Technical Report |
| **38** | United Nations International Children's Emergency Fund | 2010 | Workshop report on scaling up the use of micronutrient powders to improve the quality of complementary foods for young children in Latin America and the Caribbean | Meeting Report |
| **39** | United Nations International Children's Emergency Fund | 2009 | Workshop report on scaling up the use of multiple micronutrient powders to improve the quality of complementary foods for young children in Asia | Meeting Report |
| **40** | United States Agency for International Development | 2013 | Annual food aid program results (Wuqu’ Kawoq/Guatemala) | Annual Report |
| **41** | University of Toronto / Zlotkin, S; Aupperle, T; Grasset, C | 2008 | Research to practice: A multisectoral approach to product development, research and global distribution to prevent hidden hunger | Powerpoint slides |
| **42** | US Centers for Disease Control and Prevention / Jefferds, ME | 2014 | Designing and implementing behavior change interventions for improved adherence to micronutrient powder interventions: Lessons learnt | Powerpoint slides |
| **43** | World Food Programme | 2014 | How the Government of the Dominican Republic reduced anemia by 50% in vulnerable children, with support from WFP | Case Study |
| **44** | World Vision International | 2005 | Effectiveness of home-based fortification of complementary foods with Sprinkles in an integrated nutrition program to address rickets and anemia | Technical Report |
| **45** | World Vision International / Altengeral, S; MacDonald, C | 2011 | National scale-up of micronutrient powders in Mongolian integrated program | Powerpoint slides |

**Global Guidance on MNP Programming, by most recent**

| **No** | **Organization(s)** | **Year** | **Title** |
| --- | --- | --- | --- |
| **1** | United Nations International Children's Emergency Fund, U.S. Centers for Disease Control and Prevention, Home Fortification Technical Advisory Group | 2016 | Micronutrient powder (MNP) toolkit to support countries in implementing MNP programs |
| **2** | Home Fortification- Technical Advisory Group | 2015 | Planning for program implementation of home fortification with micronutrient powders (MNP): A step-by-step manual |
| **3** | United Nations International Children's Emergency Fund | 2015 | Home fortification, using multiple micronutrient powders: Products, procurement, and supply |
| **4** | Home Fortification- Technical Advisory Group | 2014 | MNP and the international code of marketing of breastmilk substitutes |
| **5** | Home Fortification- Technical Advisory Group | 2014 | HF-TAG quality manual on micronutrient powders – A guiding document |
| **6** | CODEX | 2013 | Revised 2013 guidelines on formulated complementary foods for older infants and young children (CAC/GL 8-1991) |
| **7** | Home Fortification- Technical Advisory Group | 2013 | HF-TAG manual on micronutrient powder (MNP) composition: Guidelines and specifications for defining the micronutrient composition of single serve sachets for specified target populations in low- and middle-income countries with high prevalence of anaemia and micronutrient deficiencies |
| **8** | Home Fortification- Technical Advisory Group | 2013 | A manual for developing and implementing monitoring systems for home fortification interventions |
| **9** | World Food Programme | 2013 | Managing the supply chain of specialized nutritious foods |
| **10** | World Health Organization | 2011 | Guideline: Use of multiple micronutrient powders for home fortification of foods consumed by infants and children 6–23 months of age |
| **11** | World Health Organization | 2010 | Indicators for assessing infant and young child feeding practices – Part II measurement |
| **12** | World Health Organization | 2010 | Indicators for assessing infant and young child feeding practices – Part III |
| **13** | World Health Organization | 2008 | Indicators for assessing infant and young child feeding practices – Part I definition |
| **14** | Sprinkles Global Health Initiative | 2008 | Micronutrient Sprinkles for use in infants and young children: Guidelines on recommendations for use and program monitoring and evaluation. |
| **15** | CODEX | 2005 | 2005 Codex guidelines for vitamin and mineral food supplements |
| **16** | Global Alliance for Improved Nutrition, Helen Keller International, Micronutrient Initiative, Sight and Life, Sprinkles Global Health Initiative, United Nations International Children’s Emergency Fund, World Food Programme for HF-TAG | Unknown | Programmatic guidance brief on use of micronutrient powders (MNP) for home fortification |
